# The mitochondrial genome of the black-tailed dusky antechinus (*Antechinus arktos*)

**DOI:** 10.1080/23802359.2020.1840940

**Published:** 2020-12-24

**Authors:** Yuepan Geng, Chen Yang, Han Guo, Patrick B. Thomas, Penny L. Jeffery, Lisa K. Chopin, Andrew M. Baker, Ran Tian, Inge Seim

**Affiliations:** a Integrative Biology Laboratory, College of Life Sciences, Nanjing Normal University, Nanjing, PR China; b Australian Prostate Cancer Research Centre-Queensland, Translational Research Institute – Institute of Health and Biomedical Innovation, Queensland University of Technology, Brisbane, Australia; c Ghrelin Research Group, Translational Research Institute –Institute of Health and Biomedical Innovation, School of Biomedical Sciences, Queensland University of Technology, Brisbane, Australia; d School of Biology and Environmental Science, Queensland University of Technology, Brisbane, Australia; e Natural Environments Program, Queensland Museum, Queensland, Australia

**Keywords:** Mitochondrial genome, carnivorous marsupial, Dasyuridae, Australia, Antechinus

## Abstract

In this study, we report the mitochondrial genome of the black-tailed antechinus (*Antechinus arktos*), a recently-discovered, endangered carnivorous marsupial inhabiting a caldera that straddles the border of Australia’s mid-east coast. The circular *A. arktos* genome is 17,334 bp in length and has an AT content of 63.3%. Its gene content and arrangement are consistent with reported marsupial mitogenome assemblies.

Here, we present the complete mitochondrial genome of the black-tailed dusky antechinus (*Antechinus arktos*). Described in 2014, *A. arktos* is a rare species, apparently limited to the highest, wettest reaches of the Tweed Volcano Caldera in mid-eastern Australia (Baker et al. 2014). In 2018, the species was federally listed as Endangered, and it was recently recognized as one of the top 20 Australian mammals most likely to go extinct in the next two decades (Geyle et al. 2018).

Genomic DNA was extracted from ear tissue (voucher specimen AA100) collected from Lamington National Park, Queensland, Australia (28.26S, 153.17E). Paired-end 350 bp-insert DNA libraries were sequenced by BGI (Hong Kong), using the BGISEQ-500 instrument, to generate ∼30× genome coverage of 100 bp paired-end reads. Raw data was processed as described in a recent ‘mitocommunication’ on *Murexia melanurus* (Tian et al. 2019).

The mitogenome was assembled as follows: 48 M reads were assembled using NOVOPlasty version 2.7.2 (Dierckxsens et al. 2017), with the *ND1* coding sequence from a partial *Antechinus flavipes* mitogenome (GenBank accession no. KJ868098) (Mitchell et al. 2014) as a seed sequence and the parameters ‘*Type = mito K-mer = 23 Genome range = 15000–19000 Variance detection = no.*’ *A. arktos* reads were present at high coverage (∼1000× across the mitogenome) and assembled to give a single contig. NOVOPlasty indicated that two adjacent nucleotides (out of 17,334) could not be accurately called (likely due to their presence within a repeat region; indicated by ‘*’ in the output FASTA). Geneious Prime version 2019.1.3 (Biomatters, Auckland, New Zealand) was used to align a further 47M reads against the assembled contig and generate a consensus genome sequence – settings ‘Majority Threshold’ and a minimum calling coverage of 100 – resolving the ambiguous nucleotides in the assembly. The genome was annotated using GenBank features of the Northern quoll (*Dasyurus hallucatus;* accession no. NC_007630). We used MARS (Ayad and Pissis 2017) to rotate the sequences to the same origin.

The final circular *A. arktos* mitochondrial genome (GenBank: MK977601) is 17,334 bp in length and has a base composition of 32.8% A, 30.5% T, 12.9% G, and 23.8% C. As in other mammals, the genome has 13 protein-coding genes (PCGs) and two ribosomal (rRNA genes). The genome shares unique features with all marsupial mitogenomes reported to date. These include a ‘ACWNY’ tRNA gene re-arrangement (Paabo et al. 1991); 21 transfer RNA (tRNA), due to a *tRNA^Lys^* pseudogene (no lysine anticodon) (Janke et al. 1994; Dorner et al. 2001); and lack of an aspartic acid anticodon, likely post-transcriptionally rescued by RNA-editing (Janke and Paabo 1993). In agreement with previous reports (Krajewski et al. 2007; Kumar et al. 2017; Mutton et al. 2019), phylogenetic analysis revealed that *Antechinus* is closely related to the genera *Phascogale* of Australia and *Murexia* of New Guinea, and *A. arktos* forms a monophyletic group along with previously reported *Antechinus* species (Figure 1). Maximum-likelihood (ML; assessed using IQ-TREE (Nguyen et al. 2015)) and Bayesian Interference (BI; assessed using MrBayes version 3.2.7 (Ronquist and Huelsenbeck 2003)), with the mtMAM+I+G4 model chosen using to the Bayesian Information Criterion) resulted in an identical tree topology.

**Figure 1.**
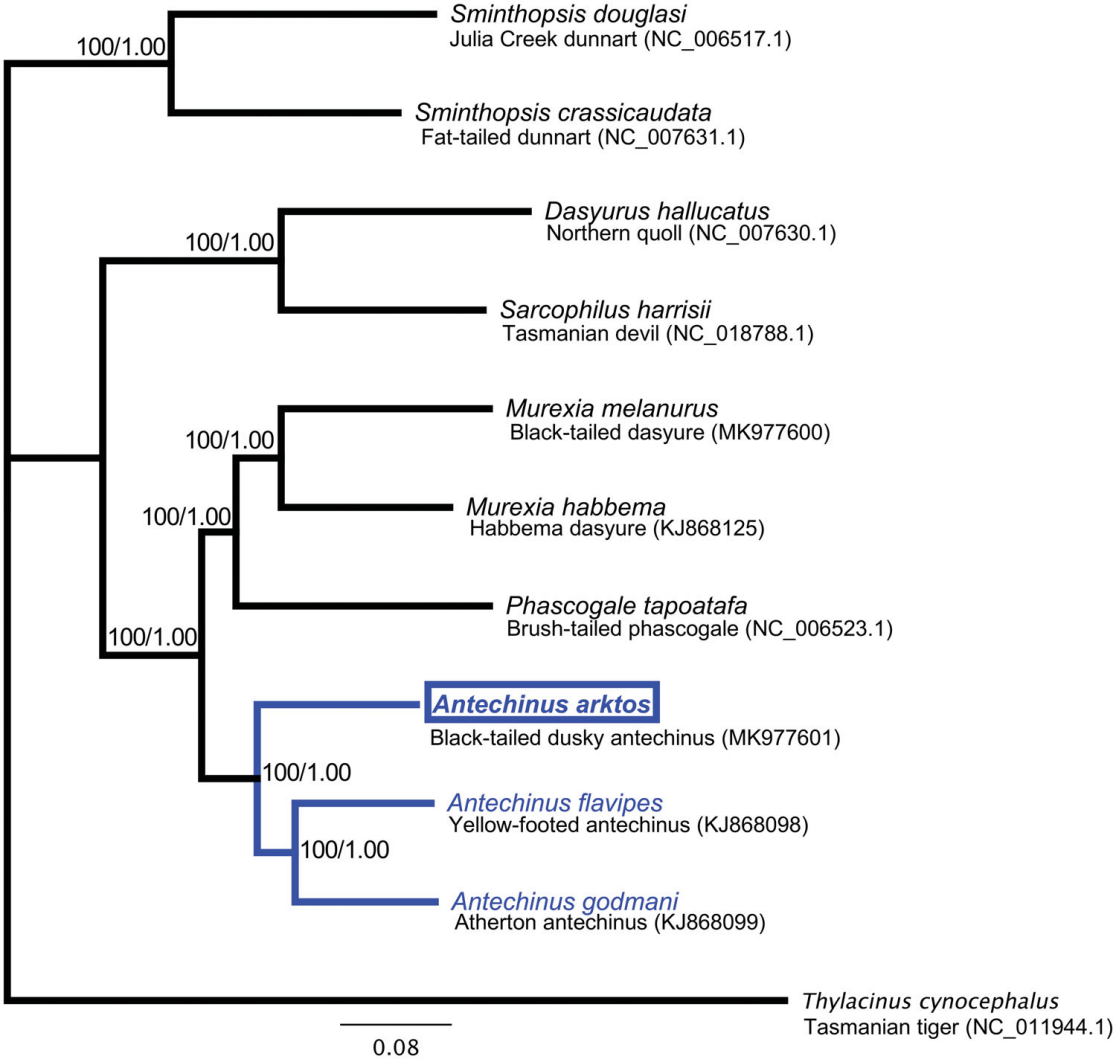
Phylogenetic tree of black-tailed dusky antechinus (*Antechinus arktos*; indicated by a box), nine other species in the marsupial family Dasyuridae, and the outgroup species *Thylacinus cynocephalus*. Phylogenetic reconstruction was performed with coding sequences of the 13 PCGs. The number at each node are ML/BI bootstrap support values.

## Geolocation information

Geospatial coordinates for the black-tailed dusky antechinus (*A. arktos*) ear tissue collection: 28.26S, 153.17E.

## Data Availability

The data that support the findings of this study is available at NCBI (National Center for Biotechnology Information) https://www.ncbi.nlm.nih.gov GenBank (accession no. MK977601).
